# Infrapopulations of *Gyliauchen volubilis* Nagaty, 1956 (Trematoda: Gyliauchenidae) in the rabbitfish *Siganus rivulatus* (Teleostei: Siganidae) from the Saudi coast of the Red Sea

**DOI:** 10.1051/parasite/2012193227

**Published:** 2012-08-15

**Authors:** M.O. Al-Jahdali

**Affiliations:** 1 Biological Sciences Department, Rabigh Faculty of Science and Arts, King Abdulaziz University P. O. Box 344 Rabigh 21911 Saudi Arabia

**Keywords:** Trematoda, *Gyliauchen volubilis*, infrapopulation, egg, mating behaviour, Red Sea fish, Trematoda, *Gyliauchen volubilis*, infrapopulation, oeuf, comportement, Mer Rouge

## Abstract

In hermaphroditic helminth parasites, infrapopulation size or mating group size mostly affects some processes acting within the infrapopulation. Here, 30 natural infrapopulations (12-154 individuals) of the intestinal trematode *Gyliauchen volubilis* Nagaty, 1956 from the fish *Siganus rivulatus* consisting of newly excysted juveniles, immature and mature worms were found distributed in a well-defined fundamental niche (anterior 40 % of the intestine). In small infrapopulations, all stages of the parasite were alive. In larger infrapopulations, differential mortality was only and consistently observed among newly excysted juveniles, and gradually increased to include most or all juveniles in the largest infrapopulations. Among mature worms, the mean worm length seemed unaffected by the infrapopulation size. However, the ratio mean testis size-mean ovary size, a reliable indicator of resource allocation to the male function and of opportunities for crossfertilization, significantly increased with mating group size. In small infrapopulations, all stages of the parasite were scattered along the niche, and never seen in mating pairs (possibly reproduced by selffertilization). In larger infrapopulations, newly excysted juveniles and immature worms were scattered along the anterior two thirds of the niche, while mature worms were constantly found aggregated in its posterior third (narrow microhabitat), where some were arranged in mating pairs. The probability of mating reciprocally or unilaterally was dependent on body size. The mean number of uterine eggs per worm significantly decreased and their mean sizes significantly increased with mating group size. The results are statistically significant and suggest that infrapopulation self-regulation is greatly associated with its size.

## Abstract (fr)

Chez les helminthes hermaphrodites parasites, la taille des infrapopulations est importante à considérer en termes de régulation. Des vers immatures et adultes de 30 infrapopulations naturelles (12-154 individus) du trématode intestinal *Gyliauchen volubilis* Nagaty, 1956, ont été examinés au niveau de l’intestin du poisson *Siganus rivulatus*. Chez les petites infrapopulations, tous les stades du parasite ont été observés vivants. Chez les plus grandes, une mortalité différentielle a été observée parmi les stades les plus jeunes, augmentant progressivement chez tous les stades adolescents avec la taille des infrapopulations. La longueur moyenne des vers à maturité n’est pas influencée par la taille de l’infrapopulation. Cependant, le ratio taille moyenne du testicule/ taille moyenne de l’ovaire diffère avec la taille de l’infrapopulation. Chez les petites infrapopulations, tous les stades du parasite sont observés tout au long de l’intestin. Chez les plus grandes, les vers adolescents les plus jeunes et les autres encore immatures sont observés dans les deux premiers tiers de l’intestin, tandis que des vers à maturité sont constamment trouvés agrégés au niveau du dernier tiers, dont certains accouplés en paires. La probabilité d’accouplement est dépendante de la taille des vers. Le nombre moyen d’oeufs intra-utérins par ver augmente de façon significative avec la taille de l’infrapopulation. Ainsi, l’autorégulation d’une infrapopulation est significativement corrélée à sa taille.

## Introduction

The parasite population is broken up into smaller units, or infrapopulations, each consisting of all conspecific parasites inside one individual host (Bush *et al.* 1997). In gastrointestinal helminth parasites, infrapopulation size or mating group size mostly affects some processes acting within the infrapopulation, such as the survival of worms, their growth, their mating probability, their fecundity, etc. (see Keymer 1982; Shostak & Scott 1993; Dezfuli *et al.*, 2002; Poulin 2007; Wedekind *et al.*, 1998; Sasal *et al.*, 2000; Al-Jahdali & Hassanine 2012a; Al-Jahdali 2011). In simultaneous hermaphroditic species, infrapopulation size or mating group size also affects the opportunities for cross-fertilization (Esch & Fernandez 1993; Trouvé *et al.*, 1999; Brown *et al.*, 2001), the ratio of resources allocated in mating to the male function versus the female function (Charnov 1982; Trouvé *et al.*, 1999; Schärer & Wedekind 2001), and the egg production (Wedekind *et al.*, 1998).

The siganid fish *Siganus rivulatus* Forsskål & Niebuhr is common in the Red Sea and is parasitized by three intestinal helminths belonging to three different phyla: the simultaneous hermaphroditic trematode *Gyliauchen volubilis* Nagaty, 1956 (Gyliauchenidae Fukui, 1929) (see Nagaty, 1956), the unisexual acanthocephalan *Sclerocollum saudii* Al-Jahdali, 2010 (Cavisomidae Meyer, 1932) (see Al-Jahdali, 2010) and the unisexual nematode *Procamallanus elatensis* Fusco & Overstreet, 1979 (Camallanidae Cobbold, 1864) (see Fusco & Overstreet, 1979). Recently, we described and analysed the infrapopulations of *S. saudii* (see Al-Jahdali & Hassanine, 2012a) and *P. elatensis* (see Al-Jahdali, 2011) when in single species infection. However, we have thus far only described the life cycle of *G. volubilis* (see Al-Jahdali & Hassanine, 2012b). In the present study, a considerable number of infrapopulations of this trematode were observed and analysed for the first time under natural conditions to assess whether infrapopulation size determines microhabitat use, survival, growth, mating behaviour and allocation to reproduction in a simultaneous hermaphroditic trematode.

I expected that, in large infrapopulations, some of the developmental stages undergo differential mortality and the growth adversely affected (density-dependent effects). I further expected that individuals that mature in a large group allocate more resources to male function than to female function to replenish the sperm used up in sperm competition.

## Materials and Methods

In a small lagoon within the mangrove swamps near Rabigh (between 22° 49’ N and 22° 54’ N) on the western Red Sea coast of Saudi Arabia, the rabbitfish fish *S. rivulatus* Forsskål & Niebuhr, 1775 (Teleostei, Siganidae) is permanently resident, and is parasitized by the intestinal trematode *G. volubilis* Nagaty, 1956. Larval forms of this trematode are found in the cerithiid gastropod *Clypeomorus clypeomorus* Jousseaume, 1888 in the same lagoon, and its life-cycle extends for about 26 weeks (see Al-Jahdali & Hassanine, 2012b). In this cycle, fully developed cercariae emerge from snail 16-18 weeks post-infection and rapidly encyst on aquatic vegetation to form metacercariae (remain alive for about one week). Metacercariae ingested by the fish excyst in the intestine into juvenile worms, develop within four-five weeks into immature worms, then develop within two-three weeks into mature worms.

Examination of numerous specimens of *S. rivulatus* from those inhabiting the coast of Yanbu (200 km north of the mangrove swamps) revealed that they were not naturally infected with any intestinal helminth parasite. Therefore, 70 specimens of nearly equal lengths (12-16 cm) were marked and transferred alive in June of 2010 to the lagoon to allow their infection with *G. volubilis* to be followed after ten weeks. To prevent fishes from escaping from the lagoon, a plastic net with narrow mesh size was used as a barrier at the lagoon mouth; the lagoon can therefore be considered as a natural aquarium. Through one week, 42 specimens (five-seven specimens/ day) of the marked fishes were caught from the lagoon by hand net. To avoid parasite postmortem or other migration along the gastrointestinal tract, each fish was killed and measured immediately after capture by a blow to the head and examined at a field laboratory (within 30-45 min after capture). Then the entire alimentary canal of each fish was immediately removed and, to record the exact position of individual parasites, the intestine was cut into ten equal sections; tied before cutting. Each section was opened and its contents examined under a dissecting stereomicroscope; individual parasites were examined to determine if they were alive or not, carefully teased out, re-examined alive in a saline solution, and the opened section was then shaken vigorously in a jar of saline to dislodge further worms and to remove mucus. Trematodes were placed in whirling hot water before fixation in hot 5% formalin under a slight coverslip pressure. This procedure gives specimens a uniform size and shape. The infrapopulation collected from each fish host was carefully counted, and its distribution in the intestine was recorded. Wholemounts were stained in alum carmine, cleared in terpineol and mounted in Canada balsam. All trematodes recovered were identified and their body lengths, round testes, round ovaries and oval eggs were measured (in micrometres) using a compound microscope with an eyepiece micrometer. The different stages of the parasite were classified according to Al-Jahdali & Hassanine (2012b); worms were categorized as newly excysted juveniles if they were closely similar to metacercariae, as immature if the sexual organs were little or moderately developed and the uterus contained no eggs, and as mature if the sexual organs were fully developed and the uterus contained eggs.

The term “mean intensity” follows the definition of Bush *et al.* (1997) and refers to the mean number of worms found per infected host. The ratio mean testis size-mean ovary size was used as a reliable indicator of resource allocation to the male function and to indicate opportunities for cross-fertilization according to Thomas and Poulin (2003) and Schärer (2009). The egg size computed using the formula of an ellipsoid as in Trouvé *et al.* (1999) (Volume = π × L × W2/6, where W is the egg width and L is the egg length). Linear regression analyses were used to determine possible relationships between data obtained (by the SPSS software, version 11.0 for Windows; SPSS, Inc., Chicago, USA).

## Results

### Infection levels and worm distribution within the intestine

Of the 42 *S. rivulatus* examined, 30 (71.4%) were slightly or heavily parasitized by the intestinal trematode *G. volubilis* Nagaty, 1956 (Digenea: Gyliauchenidae); the absence of other helminth parasites in the intestine of this fish excluded the confounding influence of interspecific interaction. A relatively large number of *G. volubilis* (2,682 specimens) belonging to 30 infrapopulations, ranging from 12 to 154 individuals, were collected from the infected fishes, with a mean intensity of 89.4 (standard deviation ± 42.2) worms/host. Because fishes were nearly equal in size, no significant relationship was found between fish size and size of *G. volubilis* infrapopulations (*R*^*2*^ = 0.035, slope = 6.793, P > 0.614). Infrapopulations were arranged according to their densities and the corresponding entire data set is shown in Tables I–IV.

**Fig. 1.**
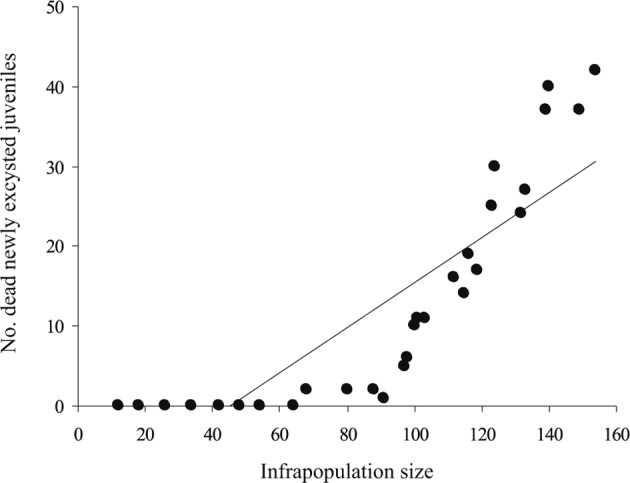
The relationship between number of dead newly excysted juveniles and infrapopulation size, in 30 infrapopulations of *Gyliauchen volubilis* (see also Table I).

*G. volubilis* infrapopulations consisted of newly excysted juveniles (coming from new infections with metacercariae), immature and mature worms. These stages were only found in the anterior four segments (40 %) of the intestine of *S. rivulatus*, *i.e.* in a welldefined fundamental niche along the intestine of this fish. In all infrapopulations, immature and mature worms were found alive, while newly excysted juveniles were found alive or dead. In small infrapopulations (I-IX, slight densities), all newly excysted juveniles were alive. In larger infrapopulations (X-XXX, higher densities), the number of dead newly excysted juveniles gradually increased with infrapopulation size to include all juveniles in the largest infrapopulations (Table I). Dead juveniles (not included in infrapopulation size) were mostly found in second segment of the intestine and some were scattered along the posterior intestine (possibly carried out by the intestinal peristaltic to this region); their body walls seemed less transparent, their internal fluids exhibited no movement and their bodies were completely immobile. The relationship between number of dead newly excysted juveniles and infrapopulation size was strongly positive (*R*^*2*^ = 0.829, slope = 3.574, *P* < 0.001) (Fig. 1), *i.e.* as the infrapopulation size increased the number of dead newly excysted juveniles significantly increased. Moreover, there were strong positive relationships between number of dead newly excysted juveniles and number of immature worms (*R*^*2*^ = 0.810, slope = 0.738, *P* < 0.001) and number of mature worms (*R*^*2*^ = 0.822, slope = 0.531, *P* < 0.001), *i.e.* as the number of immature and mature worms increased in an infrapopulation the number of dead newly excysted juveniles significantly increased.

**Fig. 2.**
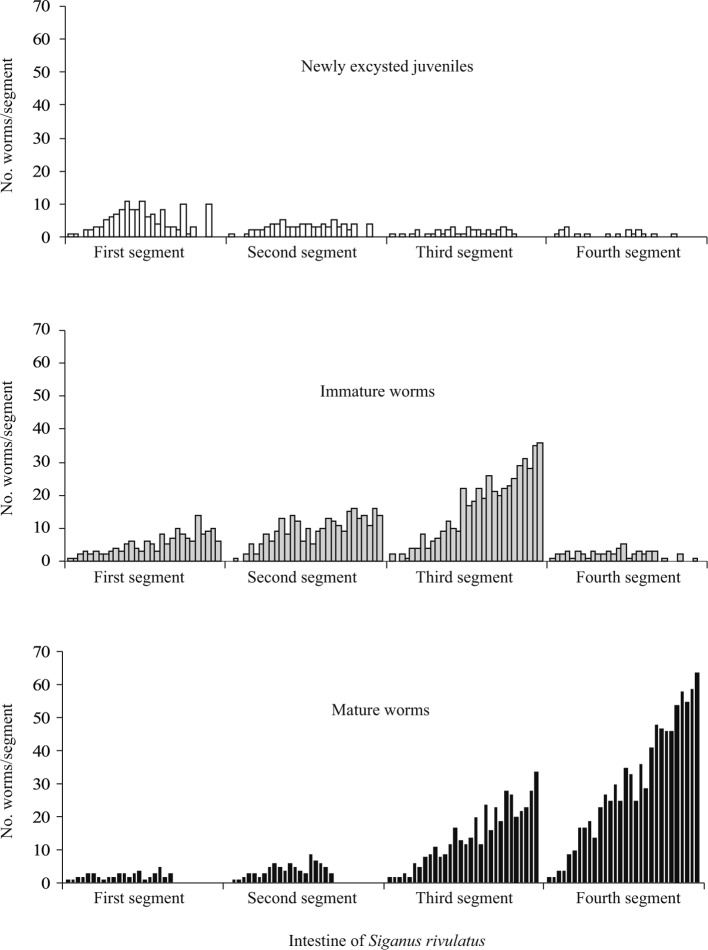
The distribution of newly excysted juveniles, immature and mature worms of *Gyliauchen volubilis* (in 30 infrapopulations; each represented by a bar) along the anterior four segments of the intestine of *Siganus rivulatus* (see also Table II).

The proportions of newly excysted juveniles (0-25%), immature worms (27.7-43.91%) and mature worms (41.6-63.6%) followed a clear ascending order in each infrapopulation (Table I). To elucidate the longitudinal distribution of these stages along the anterior four segments of the host intestine, the numbers of each stage in each segment were perfectly recorded through all infrapopulations (Table II). In small infrapopulations, the numbers were low and the stages were scattered without a clear trend along the entire length of the four segments. In large infrapopulations, the numbers were considerable and the distribution seemed to follow a definite trend (Fig. 2). In the first segment, newly excysted juveniles were the greater in number, immature worms were slightly less than them, while mature worms were few or absent (probably migrated to another segment). In the second segment, newly excysted juveniles significantly decreased in number, immature worms slightly increased, while mature worms were few or absent (probably migrated to another segment). In the third segment, newly excysted juveniles continued to decrease in number (very few or absent), while immature and mature worms continued to increase; mature worms were aggregated in the posterior third of this segment. In the fourth segment, newly excysted juveniles and immature worms were very few or absent, while mature worms continued to increase in number and aggregation. Therefore, newly excysted juveniles and immature worms were found scattered along the anterior two thirds of the niche, while mature worms were constantly found aggregated in its posterior third, *i.e.* in a narrow microhabitat. This distribution may result from recruitment dynamics, and clearly demonstrates that in large infrapopulations of *G. volubilis* the developing stages migrate towards the posterior third of their niche while they mature and reproduce.

**Table .I.** The numbers and proportions of newly excysted juveniles, immature and mature worms, in 30 infrapopulations of *Gyliauchen volubilis*.

	Newly excysted juveniles			
Infra population (No. individuals)	No. living (%)	No. dead*	Immature worms No. (%)	Mature worms No. (%)
I (12)	3 (25.0)	0	4 (33.3)	5 (41.6)
II (12)	2 (16.6)	0	4 (33.3)	6 (50.0)
III (18)	3 (16.6)	0	6 (33.3)	9 (50.0)
IV (26)	6 (23.0)	0	9 (34.6)	11 (42.3)
V (34)	5 (14.7)	0	12 (35.2)	17 (47.0)
VI (42)	8 (19.0)	0	12 (28.S)	22 (52.3)
VII (48)	5 (10.4)	0	17 (35.4)	26 (5.1)
VIII (54)	10 (18.5)	0	15 (27.7)	29 (53.7)
IX (64)	11 (17.1)	0	18 (28.1)	35 (54.7)
X (68)	13 (19.1)	2	22 (32.3)	33 (48.5)
XI (80)	14 (17.5)	2	27 (33.7)	39 (48.7)
XII (88)	17 (19.3)	2	28 (31.8)	43 (48.8)
XIII (91)	14 (15.3)	1	32 (35.1)	45 (49.5)
XIV (97)	13 (13.4)	5	29 (29.9)	55 (56.7)
XV (98)	16 (16.3)	6	36 (36.7)	46 (46.9)
XVI (100)	15 (15.0)	10	34 (34.0)	5l (51.0)
XVII (101)	13 (12.8)	11	30 (29.7)	58 (57.4)
XVIII (103)	11 (10.6)	11	37 (35.9)	55 (53.4)
XIX (112)	14 (12.S)	16	39 (34.8)	59 (52.6)
XX (115)	8 (6.9)	19	47 (40.8)	60 (52.2)
XXI (116)	10 (8.6)	14	43 (37.0)	63 (54.3)
XXII (119)	7 (5.9)	25	41 (34.4)	71 (59.6)
XXIII (123)	17 (13.8)	17	40 (32.5)	66 (53.6)
XXIV (124)	5 (4.0)	30	45 (36.3)	74 (59.6)
XXV (129)	9 (10.0)	40	47 (37.4)	73 (52.5)
XXVI (132)	0 (0)	37	58 (43.9)	74 (56.0)
XXVII (133)	0 (0)	27	53 (39.8)	80 (60.1)
XXVIII (140)	14 (10.0)	24	48 (34.3)	78 (55.7)
XXIX (149)	0 (0)	37	62 (41.6)	87 (58.4)
XXX (154)	0 (0)	42	56 (36.3)	98 (63.6)

* Not included in the infrapopulation size.

### Mean length of mature worm

**Fig. 3:**
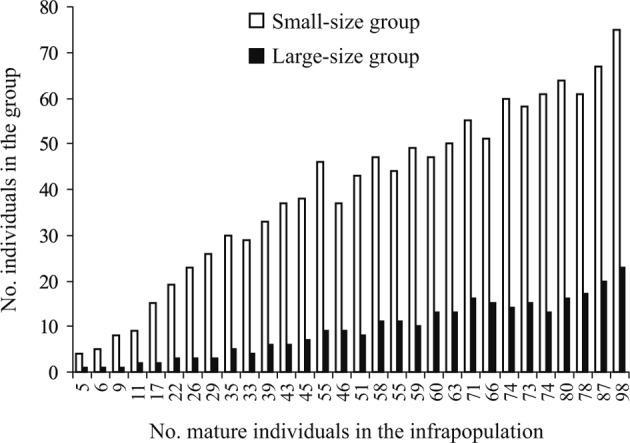
The numbers of mature worms in the small (2,361-2,811 μm in length) and large (3,182-3,775 μm in length) size groups, in 30 infrapopulations of *Gyliauchen volubilis* (see also Table III).

In infrapopulations of *G. volubilis*, mature worms ranged from 2,361 to 3,775 μm in length, and could be assigned to two size groups; a large small-size group including 4-75 worms (76.5-89.5% of mature worms) with nearly equal sizes (2,361-2,811 μm in length), and a small large-size group including 1-23 worms (10.8-23.5% of mature worms) with distinctly larger sizes (3,182-3,775 μm in length). The number and proportion of each group were recorded through all infrapopulations (Table III, Fig. 3). The relationships between numbers of worms in both groups was strongly positive (*R*^*2*^ = 0.914, slope = 3.055, *P* < 0.001). Thus, as the number of worms in the small size-group increased in an infrapopulation, the number of worms in the large size-group consequently increased. The mean worm length ranged from 2,724 to 2,990 μm through all infrapopulations. There was no significant relationship between mean worm length and both infrapopulation size (*R*^*2*^ = 0.002, slope = − 8.005, *P* = 0.892) and number of mature worms (*R*^*2*^ = 0.021, slope = − 3.005, *P* = 0.984). Thus, the mean worm length seemed to be less affected or unaffected by the infrapopulation size or by the number of mature worms.

**Table II.** The numbers of different stages of *Gyliauchen volubilis* (in 30 infrapopulations) along the anterior four segments of the intestine of *Siganus rivulatus*.

	Intestine of *Siganus rivulatus*											
	First segment			Second segment			Third segment			Fourth segment		
Infrapopulation (No. individuals)	Newly excysted juveniles	Immature	Mature	Newly excysted juveniles	Immature	Mature	Newly excysted juveniles	Immature	Mature*	Newly excysted juveniles	Immature	Mature
I (12)	1	1	1	1	0	0	1	2	2	0	1	2
II (12)	1	1	1	0	1	1	0	0	2	1	2	2
III (18)	0	2	2	0	0	1	1	2	2	2	2	4
IV (26)	2	3	2	1	2	2	0	1	3	3	3	4
V (34)	2	2	3	2	5	3	1	4	2	0	1	9
VI (42)	3	3	3	2	2	3	2	4	6	1	3	10
VII (48)	3	2	2	2	5	2	0	8	5	0	2	17
VIII (54)	5	2	1	3	8	3	1	4	8	1	1	17
IX (64)	6	3	2	4	6	5	1	6	9	0	3	19
X (68)	7	4	2	4	9	6	2	7	11	0	2	14
XI (80)	8	3	3	5	13	5	1	9	8	0	2	23
XII (88)	11	5	3	3	8	4	2	12	9	1	3	27
XIII (91)	8	6	2	3	14	6	3	10	12	0	2	25
XIV(97)	8	4	3	3	12	5	1	9	17	1	4	30
XV (98)	11	3	4	4	6	4	1	22	13	0	5	25
XVI (100)	6	6	1	4	10	3	3	17	12	2	1	35
XVII (101)	7	5	2	3	5	9	2	18	14	1	2	33
XVIII (103)	4	3	3	3	9	7	2	22	20	2	3	25
XIX (112)	8	8	5	4	10	6	1	19	12	1	2	36
XX (115)	3	5	2	3	13	5	2	26	24	0	3	29
XXI (116)	3	7	3	5	12	3	1	21	16	1	3	41
XXII (119)	2	10	0	3	11	0	2	20	23	0	0	48
XXIII (123)	10	8	0	4	9	0	3	22	19	0	1	47
XXIV (124)	1	7	0	2	15	0	2	23	28	0	0	46
XXV (129)	3	6	0	4	16	0	1	25	27	1	0	46
XXVI (132)	0	14	0	0	13	0	0	29	20	0	2	54
XXVII (133)	0	8	0	0	14	0	0	31	22	0	0	58
XXVIII (140)	10	9	0	4	11	0	0	28	23	0	0	55
XXIX (149)	0	10	0	0	16	0	0	35	28	0	1	59
XXX (154)	0	6	0	0	14	0	0	36	34	0	0	64

* Aggregated in the posterior third of the segment.

### The ratio mean testis size to mean ovary size in mature worms (mating groups)

**Fig. 4.**
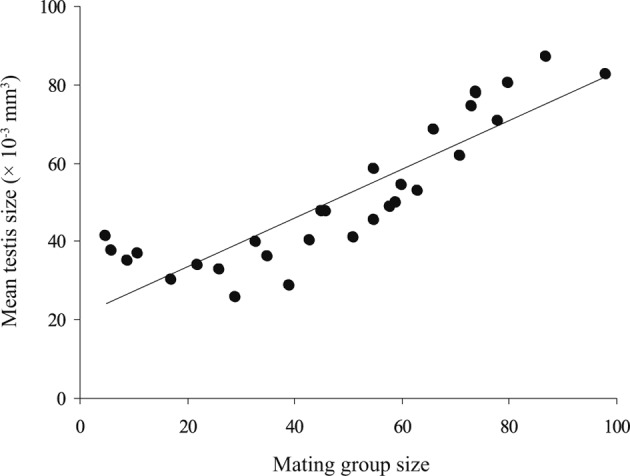
The relationship between mean testis size and mating group size, in 30 infrapopulations of *Gyliauchen volubilis* (see also Table IV).

**Fig. 5.**
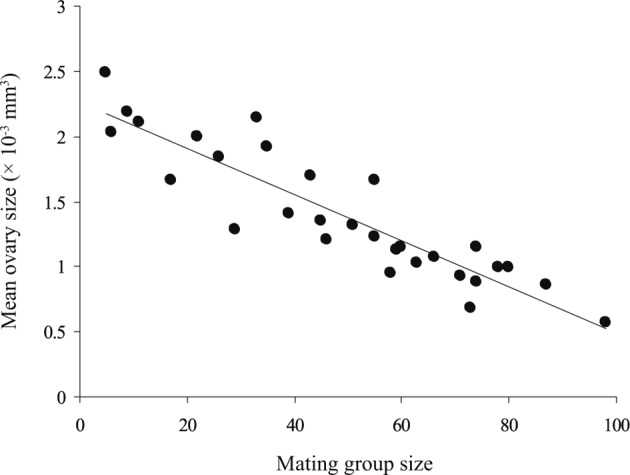
The relationship between mean ovary size and mating group size, in 30 infrapopulations of *Gyliauchen volubilis* (see also Table IV).

**Fig. 6.**
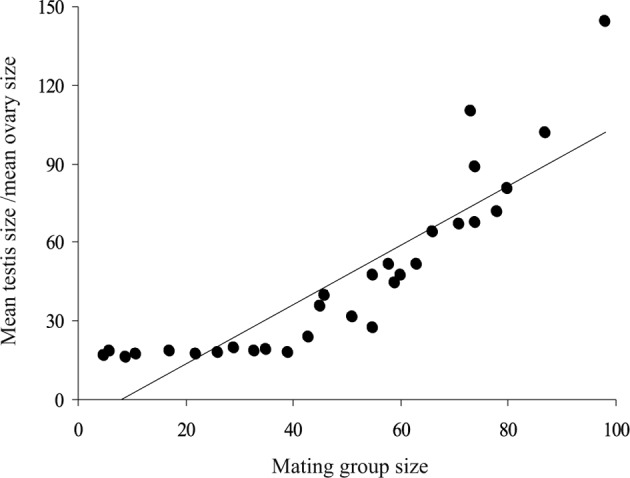
The relationship between the ratio mean testis size-mean ovary size and mating group size, in 30 infrapopulations of *Gyliauchen volubilis* (see also Table IV).

In mature worms of *G. volubilis*, the testis and ovary are round and their mean diameters ranged from 365 to 550 and from 103 to 168 μm, respectively, through the infrapopulations. The mean testis size gradually increased with mating group size (number of mature worms in infrapopulation) from 25.471 to 87.148 × 10^−3^ mm^3^, while the mean ovary size gradually decreased from 2.483 to 0.572 × 10^-3^ mm^3^. Consequently, the ratio mean testis size–mean ovary size gradually increased with mating group size from 15.91 to 144.19 (Table IV). The relationship between mean testis size and mating group size was strongly positive (*R*^*2*^ = 0.767, slope = 0.623, *P* < 0.001) (Fig. 4), while that between mean testis size and mean worm length was insignificant (*R*^*2*^ = 0.002, slope = 5.571, *P* = 0.89), *i.e.* as the mating group size increased the mean testis size significantly increased, independent of mean worm length. The relationship between mean ovary size and mating group size was strongly negative (*R*^*2*^ = 0.821, slope = − 0.017, *P* < 0.001) (Fig. 5), while that between mean ovary size and mean worm length was insignificant (*R*^*2*^ = 0.006, slope = − 0.295, *P* = 0.797), *i.e.* as the mating group size increased the mean ovary size significantly decreased, independent of mean worm length. However, the relationship between the ratio mean testis size-mean ovary size and mating group size was clearly positive (*R*^*2*^ = 0.772, slope = 0.679, *P* < 0.001) (Fig. 6). Thus, as the mating group size increased, the ratio mean testis size–mean ovary size, an indicator of resource allocation to the male function and opportunities for cross-fertilization, significantly increased, independent of mean worm length.

**Table III.** The number of individuals in small and large size groups of mature worms and the mean worm length, in 30 infrapopulations of *Gyliauchen volubilis*.

	Mature worms (mating groups)			
Infrapopulation (No. individuals)	No.	Small-size group (2,361-2,811 µm in length) No. (%)	Large-size group (3,182-3,775 µm in length) No. (%)	Mean worm length (µm) ± SD
I (12)	5	4 (80.0)	1 (20.0)	2,724 ± 114
II (12)	6	5 (83.3)	1 (16.7)	2,789 ± 103
III (18)	9	8 (88.8)	1 (11.2)	2,643 ± 78
IV (26)	11	9 (81.8)	2 (18.2)	2,622 ± 91
V (34)	17	15 (88.2)	2 (11.8)	2,770 ± 82
VI (42)	22	19 (86.3)	3 (13.7)	2,737 ± 101
VII (48)	26	23 (88.4)	3 (11.6)	2,712 ± 94
VIII (54)	29	26 (89.2)	3 (10.8)	2,681 ± 83
IX (64)	35	30 (85.7)	5 (14.3)	2,782 ± 82
X (68)	33	29 (87.8)	4 (12.2)	2,713 ± 107
XI (80)	39	33 (84.6)	6 (15.4)	2,664 ± 95
XII (88)	43	37 (86.0)	6 (14.0)	2,801 ± 103
XIII (91)	45	38 (84.4)	7 (15.6)	2,745 ± 84
XIV (97)	55	46 (83.6)	9 (16.4)	2,717 ± 93
XV (98)	46	37 (80.4)	9 (19.6)	2,783 ± 81
XVI (100)	51	43 (84.3)	8 (15.7)	2,990 ± 74
XVII (101)	58	47 (81.0)	11 (19.0)	2,808 ± 90
XVIII (103)	55	44 (80.0)	11 (20.0)	2,776 ± 77
XIX (112)	59	49 (83.0)	10 (17.0)	2,560 ± 61
XX (115)	60	47 (78.3)	13 (21.7)	2,854 ± 88
XXI (116)	63	50 (79.3)	13 (20.7)	2,621 ± 68
XXII (119)	71	55 (77.4)	16 (22.6)	2,689 ± 74
XXIII (123)	66	51 (77.2)	15 (22.8)	2,692 ± 59
XXIV (124)	74	60 (81.0)	14 (19.0)	2,637 ± 61
XXV (129)	73	58 (79.4)	15 (20.6)	2,678 ± 82
XXVI (132)	74	61 (82.4)	13 (17.6)	2,785 ± 52
XXVII (133)	80	64 (80.0)	16 (20.0)	2,794 ± 77
XXVIII (140)	78	61 (78.2)	17 (21.8)	2,706 ± 64
XXIX (149)	87	67 (77.0)	20 (23.0)	2,738 ± 67
XXX (154)	98	75 (76.5)	23 (23.5)	2,796 ± 73

**Table IV.** The mean testis size, mean ovary size, mean number of uterine eggs per worm and the mean egg size, in 30 infrapopulations of *Gyliauchen volubilis*.

	Mature worms (mating groups)							
Infrapopulation (No. individuals)	No.	Testis		Ovary		Mean testis size to mean ovary size	Mean No. uterine eggs/worm ± SD	Mean egg size (µm^3^) ± SD
		Mean diameter (µm) ± SD	Size (_10^−3^ mm^3^)	Mean diameter (µm) ± SD	Size (_10^−3^ mm^3^)			
I (12)	5	429 ± 26	41.356	168 ± 11	2.483	16.65	101 ± 9.1	49,649 ± 1,901
II (12)	6	416 ± 21	37.709	157 ± 14	2.027	18.60	98 ± 7.5	49,052 ± 1,767
III (18)	9	405 ± 19	34.796	161 ± 17	2.186	15.91	103 ± 9.2	55,777 ± 2,770
IV (26)	11	412 ± 23	36.632	159 ± 13	2.105	17.40	94 ± 7.39	51,593 ± 2,347
V (34)	17	386 ± 27	30.125	147 ± 16	1.663	18.11	96 ± 8.9	51,503 ± 2,166
VI (42)	22	401 ± 22	33.775	156 ± 17	1.998	16.90	100 ± 10.8	52,269 ± 2,744
VII (48)	26	396 ± 31	32.528	152 ± 19	1.839	17.68	80 ± 11.3	51,146 ± 2,002
VIII (54)	29	365 ± 25	25.471	135 ± 15	1.288	19.77	95 ± 9.3	56,312 ± 2,289
IX (64)	35	410 ± 36	36.101	154 ± 22	1.913	18.87	89 ± 11.3	63,678 ± 3,380
X (68)	33	423 ± 28	39.645	160 ± 18	2.145	18.48	84 ± 9.7	60,395 ± 2,990
XI (80)	39	380 ± 24	28.742	139 ± 14	1.406	17.91	74 ± 12.4	66,456 ± 2,903
XII (88)	43	425 ± 31	40.210	148 ± 16	1.698	23.68	90 ± 8.6	63,375 ± 3,178
XIII (91)	45	450 ± 34	47.732	137 ± 21	1.346	35.46	84 ± 10.3	72,589 ± 3,651
XIV (97)	55	442 ± 37	45.231	147 ± 26	1.663	27.19	88 ± 9.5	58,323 ± 3,686
XV (98)	46	449 ± 26	47.414	132 ± 21	1.204	39.38	76 ± 12.2	64,301 ± 3,244
XVI (100)	51	423 ± 32	41.068	136 ± 18	1.317	31.18	95 ± 11.0	61,589 ± 3,932
XVII (101)	58	451 ± 27	48.693	122 ± 11	0.951	51.20	73 ± 9.8	77,743 ± 4,901
XVIII (103)	55	481 ± 33	58.291	133 ± 21	1.232	47.31	82 ± 15.1	62,741 ± 4,901
XIX (112)	59	457 ± 37	49.994	129 ± 19	1.124	44.47	76 ± 13.7	68,286 ± 3,314
XX (115)	60	470 ± 32	54.383	130 ± 17	1.150	47.28	77 ± 11.5	83,041 ± 5,065
XXI (116)	63	465 ± 43	52.666	125 ± 14	1.023	51.48	62 ± 14.4	78,653 ± 3,825
XXII (119)	71	490 ± 29	61.625	121 ± 14	0.927	66.47	72 ± 12.7	73,766 ± 4,045
XXIII (123)	66	507 ± 38	68.264	127 ± 13	1.072	63.67	86 ± 8.6	81,613 ± 4,548
XXIV (124)	74	530 ± 44	77.983	129 ± 19	0.882	88.41	80 ± 10.4	87,762 ± 4,404
XXV (129)	73	522 ± 51	74.504	119 ± 16	0.678	109.88	69 ± 11.3	75,608 ± 5,094
XXVI (132)	74	529 ± 53	77.542	109 ± 19	1.150	67.42	76 ± 14.0	83,814 ± 4,517
XXVII (133)	80	535 ± 26	80.211	130 ± 22	0.998	80.37	65 ± 15.4	80,863 ± 4,280
XXVIII (140)	78	513 ± 37	70.717	124 ± 15	0.987	71.64	72 ± 12.2	81,539 ± 5,327
XXIX (149)	87	550 ± 39	87.148	118 ± 17	0.860	101.83	67 ± 12.3	91,726 ± 4,678
XXX (154)	98	540 ± 42	82.481	103 ± 21	0.572	144.19	63 ± 13.2	93,508 ± 5,227

### Mating Behaviour

In small infrapopulations of *G. volubilis*, mature worms were sluggish, scattered singly along the fundamental niche and never seen in mating pairs. In these worms, the seminal receptacle (a sperm-storage organ) was slightly swollen and faintly stained, *i.e.* slightly filled or containing a small amount of sperms. In large infrapopulations, mature worms were aggregated in a narrow microhabitat within the niche, and a considerable number of them were arranged in mating pairs. In most pairs, the two worms were from the small size-group (nearly equal in size), and when slightly separated, the cirrus of each worm was clearly seen everted (for 10-16 minutes) and the seminal receptacle of both worms was distinctly enlarged (in 126 mating pairs examined); in these pairs, insemination seemed to be reciprocal. In the other mating pairs, the two worms were distinctly differing in size (one from small size-group and one from large size-group), and when slightly separated, the cirrus of the larger worm was clearly seen everted, while that of the smaller worm was not. In addition, the seminal receptacle of the larger worm appeared to be semi-shriveled, while that of the smaller worm appeared to be greatly enlarged (in 38 mating pairs examined); in these pairs, insemination seemed to be unilateral, with the larger worm being the sperm donor and the smaller worm being the sperm recipient. Large worms were never seen together in mating pairs.

### Egg number and egg size in mature worms (mating groups)

**Fig. 7.**
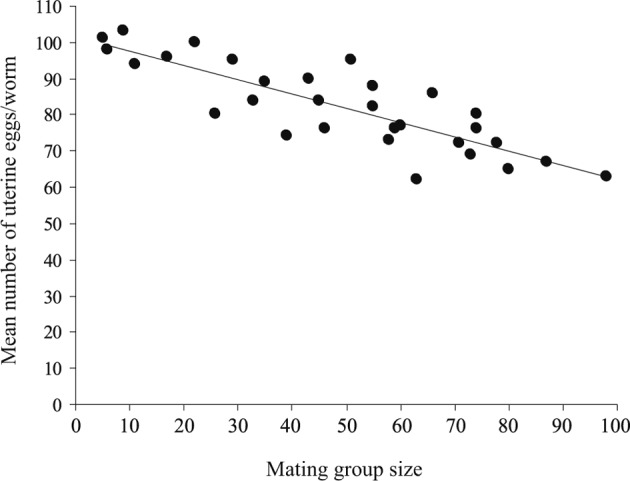
The relationship between mean number of uterine eggs and mating group size, in 30 infrapopulations of *Gyliauchen volubilis* (see also Table IV).

**Fig. 8.**
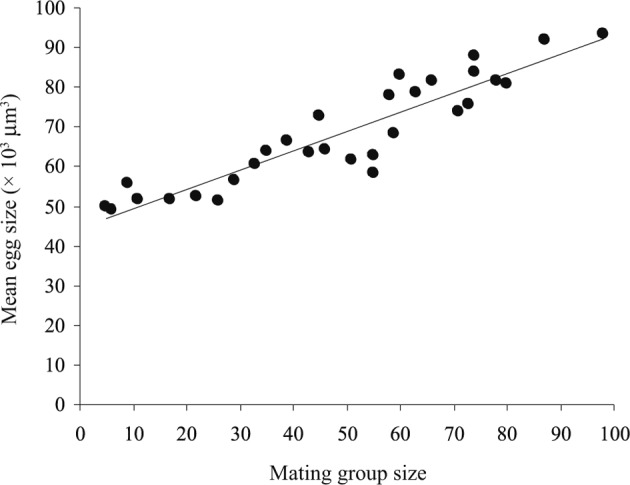
The relationship between mean egg size and mating group size, in 30 infrapopulations of *Gyliauchen volubilis* (see also Table IV).

The eggs were not observed among the intestinal contents of all infected fishes, *i.e.* the worms were not started to lay eggs at that time. The mean number of uterine eggs (per worm) gradually decreased with mating group size from 101 in the smallest mating group to 63 in the largest mating group, while the mean egg size gradually increased from 49,052 to 93,508 μm^3^, respectively (Table IV). The relationship between mean number of uterine eggs and their mean sizes was clearly negative (*R*^*2*^ = 0.635, slope = − 896.03, *P* < 0.001), *i.e.* as the mean number of uterine eggs decreased their mean sizes increased. Therefore, the relationship between mean number of uterine eggs and mating group size was significantly negative (*R*^*2*^ = 0.704, slope = − 0.393, *P* < 0.001) (Fig. 7), while that between mean number of uterine eggs and mean worm length was insignificant (*R*^*2*^ = 0.021, slope = − 10.064, *P* = 0.446). Moreover, the relationship between mean egg size and mating group size was strongly positive (*R*^*2*^ = 0.874, slope = 526.831, *P* < 0.001) (Fig. 8), while that between mean egg size and mean worm length was insignificant (*R*^*2*^ = 0.005, slope = 5303.58, *P* = 0.722). Thus, as the mating group size increased the mean number of uterine eggs decreased and their mean sizes increased, independent of mean worm length. The relationship between mean number of uterine eggs and mean ovary size was clearly positive (*R*^*2*^ = 0.654, slope = 0.035, *P* < 0.001), while that between mean egg size and mean ovary size was clearly negative (*R*^*2*^ = 0.717, slope = − 0.031, *P* < 0.001), *i.e.* as the ovary size decreased (resource allocation to the male function increased), the mean number of uterine eggs decreased and their mean sizes increased. In fact, resource allocation to eggs moved from 101 × 49,649 = 50.14 × 105 in the smallest mating group to 63 × 93,508 = 58.14 × 105 in the largest mating group. Thus, as the mating group size increases, the mean number of uterine eggs decreases but allocation per individual eggs, and total female allocation appears to increase.

## Discussion

In ecological studies of gastro-intestinal helminth parasites, the fundamental niche of a parasite is the distributional range of sites within the gut where the parasite occurs in single species infections (Poulin, 2001). In the present study, *G. volubilis* infrapopulations were found distributed in the anterior 40% of the intestine of *S. rivulatus* and never observed in the other intestinal regions. Thus, *G. volubilis* infrapopulations were distributed in a well-defined fundamental niche along the intestine of this fish; the absence of other intestinal helminth parasites excludes the confounding influence of interspecific interaction. This indicates an apparent adaptation to this site (site preference), which is probably due to physiological gradients correlated with the concentration of specific nutrients or other factors associated with fish host. In small infrapopulations, all stages of *G. volubilis* were found scattered along the entire length of the niche. In larger infrapopulations, newly excysted juveniles and immature worms were found scattered along the anterior two thirds of the niche, while mature worms were constantly found aggregated in its posterior third, *i.e.* in a narrow microhabitat.

In *G. volubilis* infrapopulations, the proportions of newly excysted juveniles, immature and mature worms showed a clear ascending order in each infrapopulation. This seemed to be normal, and was more probably due to the continuing accumulation of infections and the duration of each stage. In small infrapopulations (slight density), all stages of *G. volubilis* were found alive. In larger infrapopulations (high intensity), a differential mortality was only, and consistently, observed among the newly excysted juveniles, and significantly increased with infrapopulation size. Thus, newly excysted juveniles seemed to be adversely affected or more sensitive to crowding stress than immature and mature worms. This result strongly suggests density-dependent effects and intraspecific competition among juvenile worms and probably between them and immature and mature worms. Such a competition, which leads to individual mortality in high-density infrapopulations, may contribute to regulating the size of infrapopulations (Uznanski & Nickol 1982; Brown 1986; Kennedy 2006; Poulin 2007). However, failure of newly excysted juveniles to survive in high-density infrapopulations greatly supports the experimental finding of Uznanski & Nickol (1982) and Brown (1986) that parasite activation (excystment) is a density-independent process, but establishment and survival are apparently density dependent.

In gastrointestinal helminth parasites, density-dependent reductions in mean worm length (growth or fecundity) have been reported in several studies (*e.g.* Szalai & Dick 1989; Shostak & Scott 1993; Poulin 1999; Sasal *et al.*, 2000; Richards & Lewis 2001; Dezfuli *et al.*, 2002; Poulin *et al.*, 2003; Hassanine & Al-Jahdali 2008). Such a decrease in length (fecundity) contributes to the regulation of the parasite population by the availability of infective stages for all infrapopulations (Poulin, 2007). Unlike these studies, the mean length of mature worms of *G. volubilis* seemed to be stable or less affected through all infrapopulations, since there were no significant relationships between mean worm length and infrapopulation size, and between numbers of mature worms and their mean lengths.

In hermaphroditic helminth parasites, cross-fertilization is preferred over self-fertilization (Esch & Fernandez, 1993; Combes, 1995; Trouvé *et al.*, 1999; Brown *et al.*, 2001), suggesting that cross-fertilization may result in substantial fitness benefits for parasites (Rohde, 1994; Wedekind *et al.*, 1998; Lythgoe, 2000; Brown *et al.*, 2001). These parasites have a special mating strategy available to them in the face of sexual selection, since they can adjust the ratio of resources allocated in mating to the male function versus the female function, depending on current selection pressures and environmental conditions such as mating group size (Charnov, 1982; Raimondi & Martin, 1991; Trouvé *et al.*, 1999). However, theoretical (*e.g.*, Charnov, 1996; Lively, 1990) as well as empirical studies (*e.g.*, Wedekind *et al.*, 1998; Trouvé *et al.*, 1999; Schärer & Wedekind, 2001) revealed that the increase in resource allocation to the male function with mating group size is a consequence of local mate competition (Hamilton, 1967; Charnov, 1982), and indicates greater opportunities for cross-fertilization. These patterns were clearly observed in *G. volubilis* mating groups, where the mean testis size significantly increased and the mean ovary size significantly decreased with mating group size. However, the ratio mean testis size-mean ovary size, a reliable indicator of resource allocation to the male function and opportunities for cross-fertilization (Thomas & Poulin, 2003; Schärer, 2009) significantly increased with mating group size, and independent of the mean worm length. Moreover, mature worms in large infrapopulations were constantly found aggregated. Such aggregation increases intraspecific contact and hence facilitates cross-fertilization (Rohde, 1977). Combination of these results strongly suggests local mate competition in large mating groups or in large infrapopulations of *G. volubilis*.

In small infrapopulations of *G. volubilis*, mature worms were sluggish, scattered singly along the niche and never seen in mating pairs. Thus, mating opportunities are extremely rare, and the worms may be under strong selection for self-fertilization. According to Lüscher & Wedekind (2002), worms that seemed to avoid a potential mate either wait for a mating opportunity or reproduce by selfing. Sex allocation theory predicts that same-size animals should mate reciprocally, with pairs very different in size more likely to mate unilaterally (Angeloni *et al.*, 2002). Mating behaviour of *G. volubilis* clearly followed this pattern, since in large infrapopulations the probability of mating reciprocally or unilaterally, depended on body size. Reciprocal insemination was favored when the two worms were small (nearly equal in size) and both adopting the male role, while unilateral insemination occurred when the two worms were distinctly differing in size and only one worm (the larger one) adopted the male role. Thus, sex allocation in *G. volubilis* seemed to be size dependent, *i.e.* larger worms were more biased toward male allocation and never seen together in mating pairs. Therefore, larger worms were sperm donors and preferred mating with smaller ones (sperm acceptors).

Wedekind *et al.* (1998) studied egg production in the hermaphroditic cestode *Schistocephalus solidus* in relationship to its social situation, proving that the worm apparently adjusts its investment in eggs depending on whether the offspring is the result of self- or crossfertilization; indeed, selfers produced a larger number of eggs, but these eggs were smaller than those resulting from outbreeding individuals. To explain this variation in egg number and size, they suggested the “bet-hedging” hypothesis. A main problem of selfing is the higher frequency of genetic deficiencies among offspring due to inbreeding depression (Charlesworth & Charlesworth, 1987; Jarne & Charlesworth, 1993). Because genetic deficiencies are likely to be expressed independently of egg size, the hypothesis assumed that individuals who are forced to reproduce alone could react to this problem by spreading the risk of genetically disturbed development among more but smaller eggs, instead of producing fewer eggs, of which many fail to develop because of genetic deficiencies. The hypothesis may also provide an explanation for egg size variability in hermaphroditic helminth parasites (*e.g.*, Fenton & Hudson, 2002; Thomas & Poulin, 2003), but Schjørring (2004) disagreed, stating that these helminthes can delay and can adjust the phenology of their egg production according to the social breeding condition. In the present study, mature worms in small infrapopulations of *G. volubilis* were singly scattered along the niche, never seen in mating pairs (possibly reproduced by self-fertilization), and produced a large number of small eggs, possibly because of the high risk of genetic deficiencies due to inbreeding depression. In contrast, mature worms in large infrapopulations were aggregated, some of them were arranged in mating pairs (reproduced by cross-fertilization), and produced a smaller number of large eggs, possibly because of a lower risk of genetic deficiencies due to inbreeding depression. However, our observations during the study of the life-cycle of this trematode (see Al-Jahdali & Hassanine, 2012b) revealed that its eggs are directly ingested by the snail *C. clypeomorus*, where eggs larger than 57,235 μm^3^ in size (length × width = 62 × 42 μm) hatch in the digestive tract of the snail, while smaller eggs passed out with the snail faeces without hatching. Thus, egg production in *G. volubilis*, *i.e.* the trade-off between egg number and size, may support the bet-hedging hypothesis. In addition, this suggestion is reinforced by the negative relationship between mean number of uterine eggs (and the positive relationship between mean egg size) and mating group size, which indicate that, as the mating group size increased, the mean number of uterine eggs decreased and their mean sizes increased, *i.e.* resource allocation to egg production decreased but resource allocation to individual eggs increased, possibly related to the risk of genetic deficiencies. Generally, the exact mechanism responsible for variation in egg number and size requires genetic studies as suggested by Schjørring (2004).
